# Development of a Multi-Target Strategy for the Treatment of Vitiligo via Machine Learning and Network Analysis Methods

**DOI:** 10.3389/fphar.2021.754175

**Published:** 2021-09-15

**Authors:** Jiye Wang, Lin Luo, Qiong Ding, Zengrui Wu, Yayuan Peng, Jie Li, Xiaoqin Wang, Weihua Li, Guixia Liu, Bo Zhang, Yun Tang

**Affiliations:** ^1^ Shanghai Key Laboratory of New Drug Design, School of Pharmacy, East China University of Science and Technology, Shanghai, China; ^2^ Key Laboratory of Xinjiang Phytomedicine Resources of Ministry of Education, School of Pharmacy, Shihezi University, Shihezi, China; ^3^ Key Laboratory of Medicinal and Edible Plants Resources Development of Sichuan Education Department, Sichuan Industrial Institute of Antibiotics, School of Pharmacy, Chengdu University, Chengdu, China

**Keywords:** kaempferide, machine learning, melanogenesis, multi-target strategy, network analysis, vitiligo

## Abstract

Vitiligo is a complex disorder characterized by the loss of pigment in the skin. The current therapeutic strategies are limited. The identification of novel drug targets and candidates is highly challenging for vitiligo. Here we proposed a systematic framework to discover potential therapeutic targets, and further explore the underlying mechanism of kaempferide, one of major ingredients from *Vernonia anthelmintica (L.) willd*, for vitiligo. By collecting transcriptome and protein-protein interactome data, the combination of random forest (RF) and greedy articulation points removal (GAPR) methods was used to discover potential therapeutic targets for vitiligo. The results showed that the RF model performed well with AUC (area under the receiver operating characteristic curve) = 0.926, and led to prioritization of 722 important transcriptomic features. Then, network analysis revealed that 44 articulation proteins in vitiligo network were considered as potential therapeutic targets by the GAPR method. Finally, through integrating the above results and proteomic profiling of kaempferide, the multi-target strategy for vitiligo was dissected, including 1) the suppression of the p38 MAPK signaling pathway by inhibiting CDK1 and PBK, and 2) the modulation of cellular redox homeostasis, especially the TXN and GSH antioxidant systems, for the purpose of melanogenesis. Meanwhile, this strategy may offer a novel perspective to discover drug candidates for vitiligo. Thus, the framework would be a useful tool to discover potential therapeutic strategies and drug candidates for complex diseases.

## Introduction

Vitiligo is an acquired depigmenting skin disease due to abnormal melanocyte function, which affects 0.5–2% of the world population (Picardo et al., 2015). The loss of pigment can be a serious psychological burden for patients. Recent studies have indicated that several factors including autoimmunity (Speeckaert et al., 2017; Tulic et al., 2019) and oxidative stress (Cui et al., 2019; Yi et al., 2019) are implicated in the pathogenesis of the disease. Genome-wide association studies (GWAS) of vitiligo have identified approximately 50 different susceptibility loci (Spritz and Andersen, 2017), and revealed that it is a complex disease network regulated by immunomodulatory factors, apoptotic and melanogenic proteins (Jin et al., 2016). Although these studies can help us to understand the molecular mechanism of vitiligo, there are still great difficulties in the development of anti-vitiligo drug discovery.

Current therapeutic drugs of vitiligo focus on skin repigmentation in a way of phenotypic intervention, such as immunomodulators (glucocorticoids) and calcineurin inhibitors (tacrolimus and pimecrolimus) (Iannella et al., 2016). However, these drugs are not satisfactory to many patients because of time-consuming and adverse reactions (Picardo et al., 2015; de Menezes et al., 2016; Iannella et al., 2016). One possible reason is that these therapeutic drugs are single-target treatments, rather than multi-target strategy under the network pharmacology (Hopkins, 2008). Furthermore, traditional Chinese medicines (TCMs) have been frequently used in the treatment of vitiligo, such as *Vernonia anthelmintica (L.) willd* (Wang et al., 2017; Dogra et al., 2020). It has been reported that the *Vernonia anthelmintica (L.) willd* injection, the main ingredients of which is flavonoids such as kaempferide, has a significant therapeutic effect for vitiligo patients (Zhou et al., 2012; Huo et al., 2017; Niu et al., 2017; Lai et al., 2021). However, the multi-target mechanism of these TCM ingredients is still unclear, which severely hinders the application of TCMs in the treatment of vitiligo. Thus, there is an urgent demand to employ a systematic approach to explore the multi-target mechanism of TCM ingredients, and further discover novel therapeutic strategies.

Over the past decade, machine learning methods have been widely used in drug discovery and development (Vamathevan et al., 2019). The random walk-based method was used to explain disease treatment mechanisms based on the multiscale interactome network (Ruiz et al., 2021). Random forest (RF) was used for dimensionality reduction and classification of multi-omics data, and extensively applied for the diagnostic and therapeutic clues of complex diseases (Fortino et al., 2020). For example, a RF model was used to identify the proteomic and metabolomic characterization from 18 non-severe and 13 severe COVID-19 patients, which might be useful for prioritizing therapeutic strategies (Shen et al., 2020).

Meanwhile, network-based analysis methods were used to study the complexity of biological systems (Barabási and Oltvai, 2004; Harrold et al., 2013; Fotis et al., 2018). For example, the network-based proximity method was used to predict novel biological associations (Guney et al., 2016; Cheng et al., 2018; Cheng et al., 2019), and had been applied to drug discovery for COVID-19 (Zhou et al., 2020a; Zhou et al., 2020b) and Alzheimer’s disease (Peng et al., 2020; Zhou et al., 2021). In addition, another network analysis method was proposed by the local tree approximation theory and used to analyze articulation points (APs) in a network (Tian et al., 2017). These APs play important roles in ensuring the robustness and connectivity of many real-world networks including human disease networks. If these nodes are removed from the disease networks, these networks may be quickly decomposed. It means this method can identify key targets in disease networks.

**FIGURE 1:**
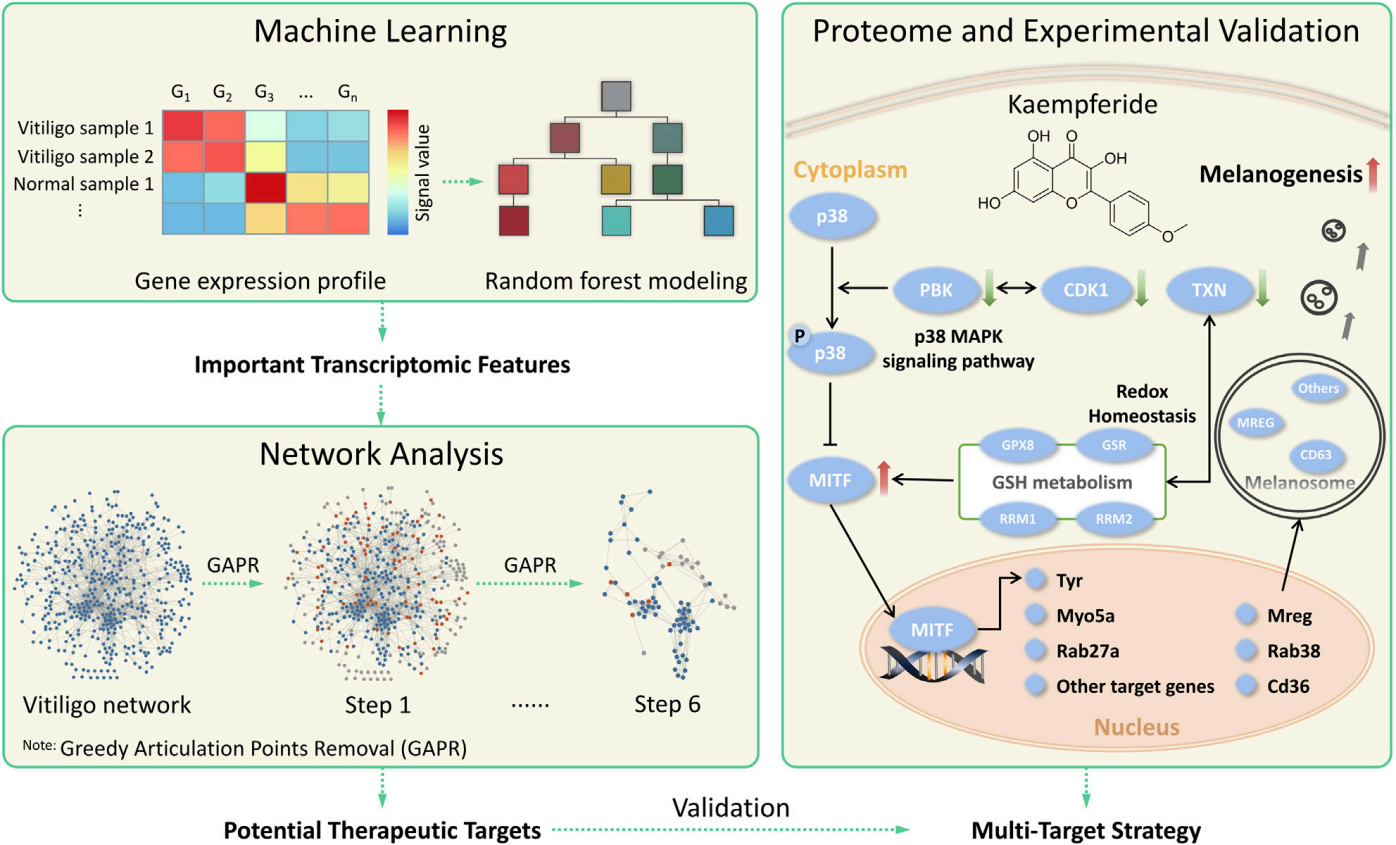
The systematic framework to discover potential therapeutic strategies for vitiligo by combining machine learning and network analysis together.

In this study, we proposed a systematic framework (Figure 1) to discover potential therapeutic targets for vitiligo via combining machine learning and network analysis together, and further explore the underlying mechanism of kaempferide. The workflow mainly included three major parts. Starting from gene expression profiles of normal and vitiligo skin samples, a random forest model was built to identify the discrimination and select important transcriptomic features for vitiligo. Then, the vitiligo protein-protein interaction subnetwork (VitNet) was constructed, and potential therapeutic targets in the VitNet were predicted by a network-based analysis method. Finally, some potential therapeutic targets were validated by proteome and experiments analysis, which was useful to explore the multi-target strategy for vitiligo.

## Materials and Methods

### Data Collection and Preparation

#### Gene Expression Dataset

Three gene expression profiles (GSE53146, GSE65127 and GSE75819) of skin samples were downloaded from Gene Expression Omnibus (GEO) database (https://www.ncbi.nlm.nih.gov/geo/). The array data of GSE53146 included five normal skin samples and five vitiligo lesional skin samples (Rashighi et al., 2014). The array data of GSE65127 consisted of 10 normal skin samples, 10 non-lesional skin samples and 10 vitiligo lesional skin samples (Regazzetti et al., 2015). The array data of GSE75819 contained 15 non-lesional skin samples and 15 vitiligo lesional skin samples (Singh et al., 2017). In this study, these non-lesional skin samples were considered as normal skin samples. The robust multiarray average (RMA) method (McCall et al., 2010) was applied to preprocess these gene expression profiles, mainly including background correction and data normalization. According to the platform information (GPL570, GPL6884 and GPL14951) of each gene expression profile, the intersection of these gene probes was considered as the common transcriptomic signatures.

#### Human Protein-Protein Interactome Dataset

In previous studies, Cheng et al. assembled 15 commonly used databases, focusing on high-quality protein-protein interactions (PPIs) with various types of evidence, to build an unbiased, systematic human protein-protein interactome dataset (Cheng et al., 2018; Cheng et al., 2019). Moreover, the STRING database (v11.0, https://string-db.org/) stored the multi-species protein-protein association networks by collecting all publicly available sources of PPIs (Szklarczyk et al., 2018; Szklarczyk et al., 2020). In this study, the above two datasets were integrated into a more comprehensive human protein-protein interactome dataset. For the STRING database, the organism *Homo sapiens* was chosen and the interaction score was set to be not less than 0.9. These protein-coding genes were then mapped to their official gene symbols and Entrez ID based on NCBI Gene database (https://www.ncbi.nlm.nih.gov/gene/). Self-interacting proteins were removed in the human protein-protein interactome dataset.

### Machine Learning

#### Random Forest

The RF (Liaw and Wiener, 2002) method was employed to build a machine learning model. We randomly split the gene expression dataset into a training set and a test set in a ratio of 8:2 for model building and validation, respectively. In the RF analysis, 500 trees were built using R package randomForest (v4.6.14) (Breiman, 2001) with 10-fold cross validation, and this was repeated for 100 times. The test set was used to evaluate the trained model. The normalized additive predicting probability was computed as the final predicting probability. The larger probability for the binary classification was adopted as the predictive label. The area under the receiver operating characteristic curve (AUC) was calculated, and the value of AUC ranges from 0.5 to 1, which represented random classifier and perfect classifier, respectively.

#### Feature Selection

Feature selection was performed based on mean decrease in accuracy (MDA) provided by the trained RF model. This method is to change the value of a variable into a random value. Then, the degree of decrease in the prediction accuracy of RF model is further evaluated. The larger MDA indicates the greater importance of the variable. In this study, those variables with MDA larger than 1 were considered as important transcriptomic features for vitiligo (Lu et al., 2020; Monforte et al., 2021).

### Network Analysis

#### Construction of the Vitiligo PPI Subnetwork

In this study, the VitNet was constructed to evaluate the functional interactions among those important transcriptomic features based on the integrated human protein-protein interactome dataset. If no interactions, these transcriptomic features (nodes) were removed from the networks. The Cytoscape software (v3.7.2) was utilized for the visualization of PPI network (Shannon et al., 2003). The NetworkAnalyzer (Assenov et al., 2007) tool was applied to compute topological parameters of the VitNet. The Molecular Complex Detection (MCODE, v1.4.2) (Bader et al., 2003) was conducted to screen core modules of the VitNet. In detail, cutoff values were 2 for the degree and 0.2 for the node score, while the k-score was 2 and the maximum depth was 100.

#### Greedy Articulation Points Removal Method

The GAPR method was developed to find APs in networks (Tian et al., 2017). This method can analyze all the APs of the initial and disrupted network based on the local tree approximation theory. A node in a network is defined as AP if its removal disconnects the network or increases the number of network-connected components. These APs can ensure the robustness and connectivity of many real-world networks including human disease networks (Tian et al., 2017). Thus, we used the GAPR method to comprehensively and deeply understand the VitNet, leading to suggestion of novel potential therapeutic targets for vitiligo.

The process of APs prediction can be considered as the network decomposition process. For an initial network (the first layer), these APs and the edges linked to them were removed from the network and formed a new disrupted network (the second layer). Then, the disrupted network was peeled off step by step, until there is no AP left and a well-defined residual giant bicomponent (RGB) is formed. The RGB was the core module of networks. These APs of each layer were regarded as articulation proteins in disease networks. And some articulation proteins were used for experimental validation.

#### Robustness Evaluation

To explore the robustness of the GAPR method, we tested if our results were tolerant against data incompleteness (Kovács et al., 2019). These PPIs of the VitNet were randomly removed at a rate of 1–50%, and this was repeated for 100 times. Then, the GAPR method was used to find articulation proteins in the incomplete VitNet. Next, the articulation protein overlap rate was calculated by comparing the differences of articulation proteins in the complete and incomplete VitNet. Finally, the robustness was represented by the mean value of articulation protein overlap rate.

### Experimental Validation

#### Chemicals and Reagents

Kaempferide (PubChem CID: 5281666, purity ≥98%), 8-MOP (PubChem CID: 4114, purity ≥98%), phenylthiourea (PTU; PubChem CID: 676454, purity ≥98%) and alpha-melanocyte-stimulating-hormone (α-MSH; PubChem CID: 16132636, purity ≥97%) were purchased from Yuanye Biotechnology (Shanghai, China). Flavopiridol (PubChem CID: 5287969, purity ≥99%) and OTS-964 (PubChem CID: 89675898, purity ≥99%) were purchased from Topscience (Shanghai, China). Dulbecco’s modified Eagle’s medium (DMEM) and First Strand cDNA Synthesis Kit was purchased from Thermo Fisher Scientific (Carlsbad, United States). Fetal bovine serum (FBS) was obtained from Biological Industries (Cromwell, United States). Rotor-Gene SYBR Green PCR Kit was purchased from Qiagen (Hilden, German). Total RNA Extractor (Trizol) kit was obtained from Sangong (Shanghai, China). Penicillin/streptomycin was purchased from Solarbio (Beijing, China).

#### *In vitro* Melanogenic Assay in B16F10 Cells

The B16F10 murine melanoma cell line was purchased from the Cancer Cell Repository (Shanghai Cell Bank, China). Cells were cultured in DMEM containing 10% FBS and 1% penicillin/streptomycin in a 5% CO_2_ humidified incubator (Thermo Fisher Scientific, United States) at 37°C. The B16F10 cells were seeded in a density of 5 × 10^4^ cells/ml in a 2 ml system and treated with DMEM, α-MSH (10 nM), flavopiridol (10 nM) and OT-964 (10 nM) for 48 h. The samples were digested and collected in 1.5 ml EP tubes, centrifuged at 1200 RPM for 10 min and photographed by the PENTAX K-7 (Tokyo, Japan).

#### *In vivo* Melanogenic Assay in Zebrafish

Adult TU zebrafish (RRID: ZIRC_ZL784) were purchased from YSY Biotechnology (Nanjing, China) and maintained in a 3 L polystyrene aquarium tank (10 zebrafish per tank) under standard conditions at 28.5°C with a 14 h light/10 h dark cycle (Lim et al., 2019). Embryos were obtained from natural crosses between the adult TU zebrafish and raised in embryonic water. Synchronized 72 h post-fertilization (hpf) embryos that were placed in 6-well plates (5 ml embryonic water; six embryos per well), were pre-treated with PTU (200 μM) for 48 hpf. PTU, a highly potent tyrosinase inhibitor, showed the strongest anti-melanogenesis effect at standard concentrations of 200 μM in zebrafish embryos. Then, these embryos were treated with 8-MOP (100 μM), flavopiridol (10 nM) and OTS-964 (10 nM) for 48 hpf. 8-MOP was used to repigment the lesional skin of vitiligo patients. Embryos were anaesthetized in clove oil and mounted in 1% methyl cellulose. The pigmentation of embryos was photographed under the OPTEC SMZ-T_2_ stereomicroscope (Chongqing, China).

#### RNA Extraction, cDNA Synthesis and qPCR

The B16F10 cells were collected to perform the qPCR analysis. The total cellular RNA was isolated using a Total RNA Extractor (Trizol) kit. RNA quality was tested using the A260/A280 ratio. The cDNA synthesis was performed using Moloney murine leukemia virus reverse transcriptase with a First Strand cDNA Synthesis Kit (Thermo Fisher Scientific, United States). The cDNA synthesis system was performed according to the manufacturer’s instructions. The abundance of *Mitf* (Microphthalmia-associated transcription factor), *Tyr* (Tyrosinase), *Gsr* (Glutathione reductase, mitochondrial) and *Gapdh* (Glyceraldehyde-3-phosphate dehydrogenase) mRNA in the samples were quantified using SYBR Green-based Rotor-Gene Q (Qiagen, German) and quantified using the 2^−ΔΔCt^ method. The mRNA expression was normalized using *Gapdh* as an endogenous control. The amplification was performed for 36 cycles (denaturing at 95°C for 10 min, annealing at 95°C for 5 s, and extension at 60°C for 45 s). These primers sequences (Supplementary Table S1) were synthesized by Sangong Co., Ltd. (Shanghai, China).

#### Proteome Analysis

The B16F10 cells were incubated with kaempferide (32 μM) for 24 h, 48 h. At the end point of this treatment, cells were collected to perform the tandem mass tag (TMT) labeling-based quantitative proteomic analysis. These samples were lysed in 300 µl lysis buffer supplemented with 1 mM PMSF (Sigma, United States) by ultrasonication. The cell lysate was 15,000 g centrifuged at 4°C for 15 min, and the supernatant was collected. Protein concentration was determined by using the BCA Protein assay (Beyotime, China) according to the manufacturer’s instructions.

The proteins were digested by trypsin through the Filter Aided Sample Preparation (FASP) method as described before (Wiśniewski et al., 2009). For TMT labeling, these samples were re-suspended in 100 μl 50 mM TEAB and 40 μl of each sample were transferred into new tubes for labeling. Subsequently, TMT labeling of individual protein digests from each sample with the TMT label reagent (Thermo Fisher Scientific, United States) was conducted according to the manufacturer’s instructions. Finally, the labeling peptides solutions of each samples were performed on the Reverse-phase liquid chromatography (RPLC)-Mass spectrometry (MS) analysis. The detail of the RPLC-MS analysis was described in Supporting Information.

The raw data were searched by the Proteome Discoverer TM 2.2 software (Thermo Fisher Scientific, United States) with the following parameters: sample type was set as TMT 10 plex (Peptide Labeled) and trypsin digestion. The Cysteine Alkylation was set as Iodoacetamide. For protein quantification method, the false discovery rate (FDR) was calculated by the target-decoy mode. The identification result was strictly filtered with 1% FDR. The differentially expressed proteins (DEPs) were characterized according to the following criteria: FC ≥ 1.5 or FC ≤ 2/3 and *p*-value < 0.05 (Student’s t-test).

#### Glutathione Species Analysis

A pair of molecularly imprinted polymer (MIP) modified electrochemical sensors (GSH-MIP and GSSG-MIP sensors) (Zhang et al., 2016) were used to detect glutathione (GSH) and glutathione disulfide (GSSG) in B16F10 cells. Both of sensors exhibited the relatively wide linear detection range and low detection limit. The B16F10 cells were treated with kaempferide (8, 16, 32 μM) for 12 and 24 h. At the end point of this treatment, these samples were frozen rapidly and thawed twice with liquid nitrogen at 37°C and then at 4°C for 5 min. The supernatant was harvested by centrifugation at 10,000 g for 10 min for glutathione species analysis.

### Bioinformatics and Statistical Analysis

The bioinformatics analysis including gene ontology (GO) and KEGG pathway enrichment analysis was performed by R package clusterProfiler (v3.16.0) (Yu et al., 2012). The statistical analysis in this study was carried out by the R software (v3.6.3). The R package pheatmap (v1.0.12) (Kolde, 2018) was applied to construct the heat map for DEPs. All values are presented as means ± standard error and analyzed by Student’s t-test and one-side Wilcoxon rank sum test. All experiments were done at least 3 times with similar results.

## Results

### Statistics of Datasets

In this study, a total of 70 skin samples (Supplementary Table S2) were collected including 40 normal skin samples (Control group) and 30 vitiligo lesional skin samples (Vitiligo group). After preprocessing and standardization of all gene expression profiles, there were a total of 11,095 transcriptomic signatures. Furthermore, we built a more comprehensive human protein-protein interactome dataset by integrating two publicly available PPIs datasets. Cheng’s PPI dataset included 217,160 PPIs connecting 15,970 unique proteins. In the STRING database, the human protein-protein interactome dataset included 489,764 PPIs connecting 11,913 unique proteins. After integration, the comprehensive human protein-protein interactome included 427,997 unique PPIs connecting 17,143 unique proteins (Supplementary Table S3).

### Important Transcriptomic Features for Vitiligo via Machine Learning

**FIGURE 2:**
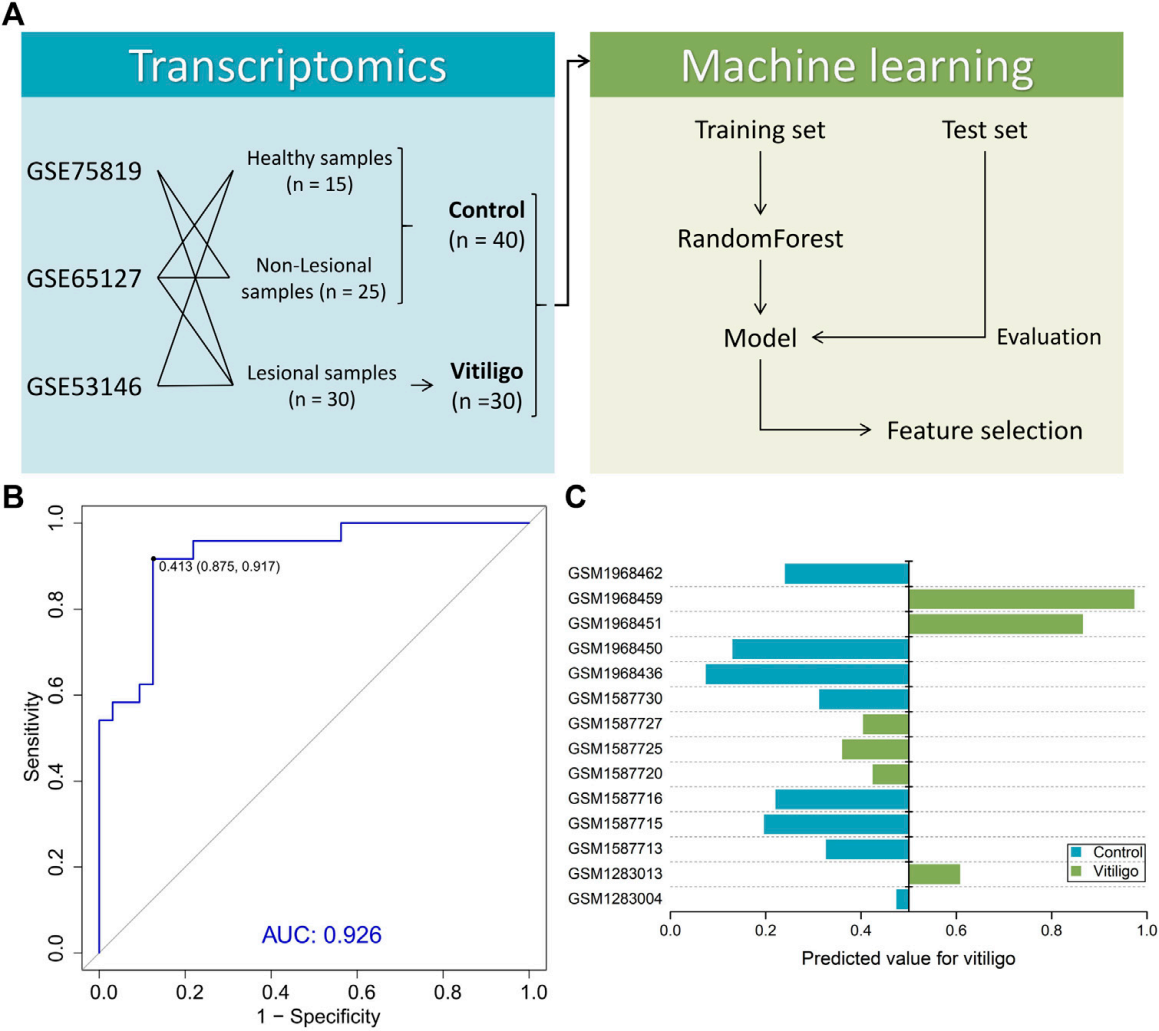
Summary of machine learning design and model performance. **(A)** Study design for machine learning-based classifier development for vitiligo patients. **(B)** Receiver operating characteristic (ROC) of the random forest model in the training set. **(C)** Performance of the model in the test set containing six vitiligo and eight normal skin samples.

We next investigated the possibility of differentiating vitiligo and normal samples from the transcriptome level. By collecting the transcriptomic data (Figure 2A), the RF method was used to build a machine learning-based classifier model for identifying the discrimination between normal and vitiligo skin samples. This model reached an AUC of 0.926 in the training set (Figure 2B; Supplementary Figure S1). We then tested the model on test set (Figure 2C). The model achieved an overall accuracy of 78.6% in the test set. All normal samples and half of vitiligo samples were correctly identified, except these three vitiligo samples (GSM1587720, GSM1587725 and GSM1587727) from the same gene expression profile (GSE65127). More samples data could improve the model performance, but an advantage of the RF method was that it could extract effective information from relatively small datasets (Shen et al., 2020; Unger et al., 2020).

**FIGURE 3:**
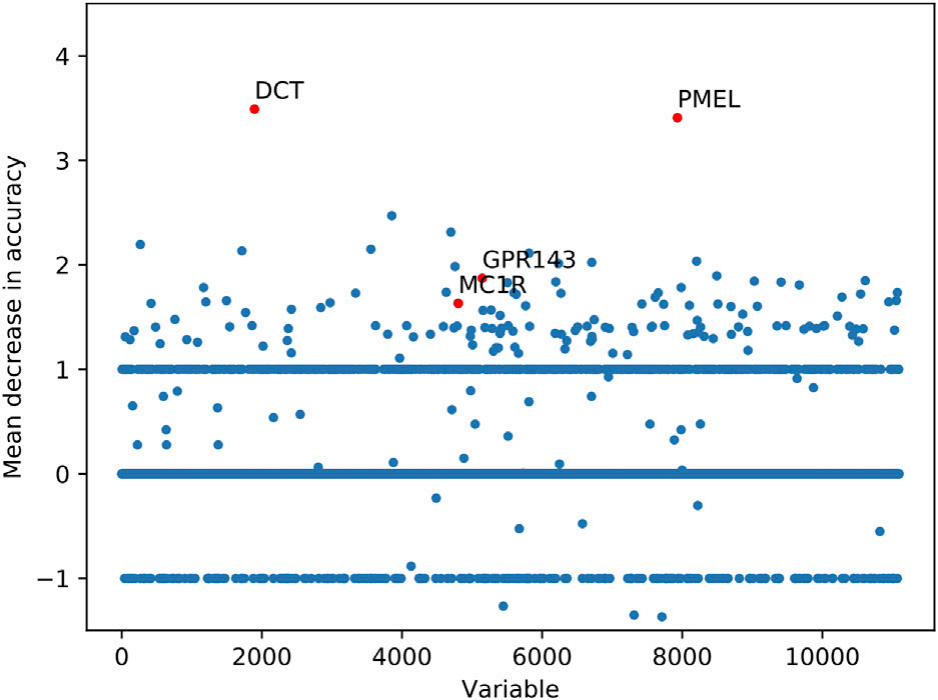
Important transcriptomic features for vitiligo prioritized by random forest analysis.

Among 11,095 variables, 722 variables with MDA larger than 1 were considered as important transcriptomic features for vitiligo based on the RF model (Figure 3). Several top-ranked variables such as DCT (L-dopachrome tautomerase), PMEL (Melanocyte protein PMEL), GPR-143 (G-protein coupled receptor 143) and MC1R (Melanocyte-stimulating hormone receptor) were closely related to the melanin-biosynthetic process. Some transcriptomic features were also associated with the immune characteristics, such as LARP7 (La-related protein 7), PBK (Lymphokine-activated killer T-cell-originated protein kinase), ABL1 (Tyrosine-protein kinase ABL1) and inflammatory cytokines (IL-19, IL-33 and IL-34) (Supplementary Table S4). These transcriptomic features may be screened as candidate vitiligo-related biomarkers and would be helpful for understanding the molecular mechanism of vitiligo and giving us the ability to discover potential therapeutic targets.

### Potential Therapeutic Targets for Vitiligo via Network Analysis

**FIGURE 4:**
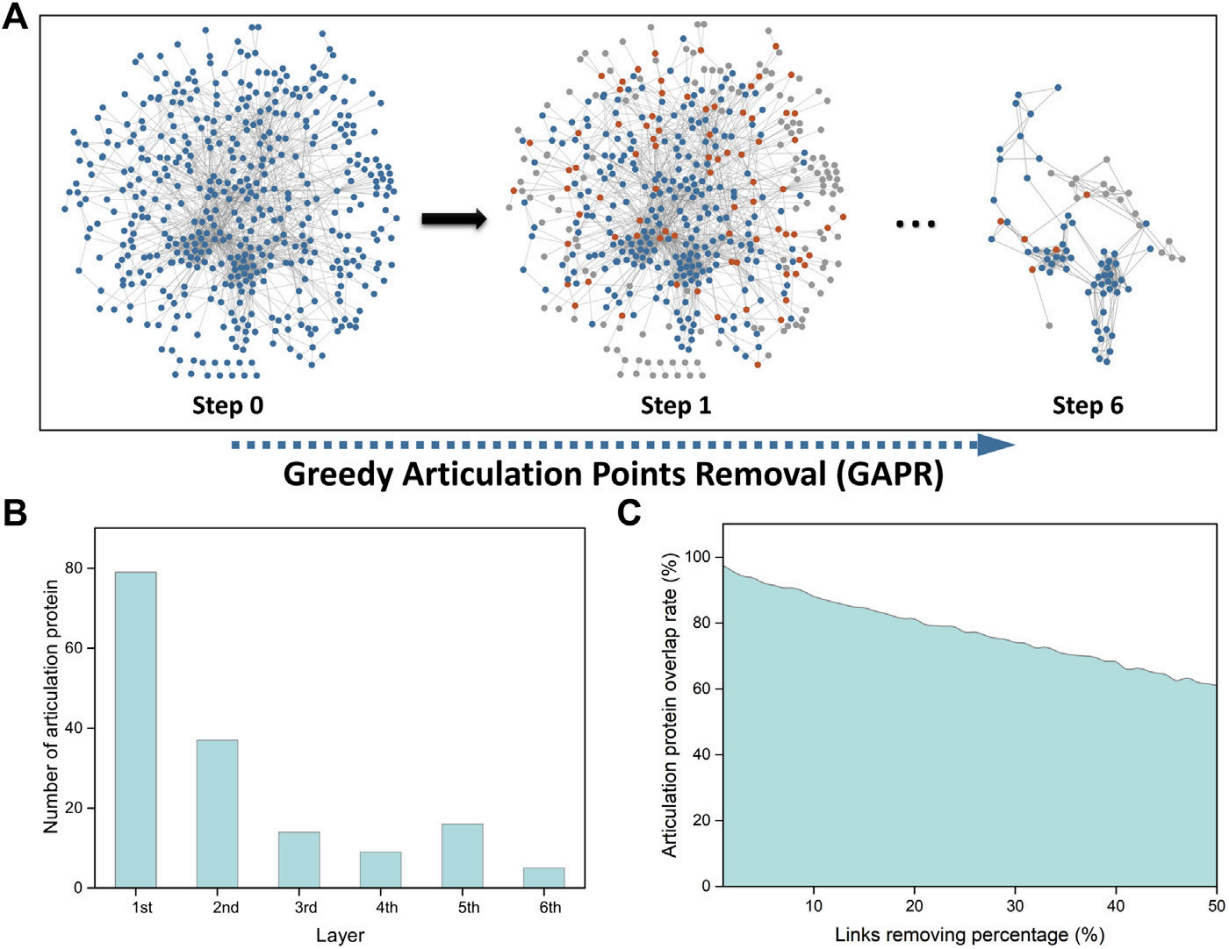
Network analysis of important transcriptomic features using greedy articulation point removal (GAPR) method. **(A)** Construction of the vitiligo PPI subnetwork (VitNet) and the GAPR method process. Blue nodes represented important transcriptomic features. Orange nodes represented articulation points. Grey nodes represented supporting points. **(B)** The number of articulation protein in the initial and deep network. **(C)** Robustness analysis of the GAPR method.

For further analysis of potential therapeutic targets for vitiligo from these important transcriptomic features, the VitNet (Figure 4A) was constructed based on the integrated human protein-protein interactome dataset, which consisted of 466 nodes and 1,583 edges (Supplementary Table S5). The node degree distribution (Supplementary Figure S2) of the VitNet followed the power law (*R* ^*2*^ = 0.920; Correlation = 0.955) indicating that the network was a scale-free network.

We then investigated that AP-based removal strategy was an effective strategy for discovery of potential therapeutic targets. When these APs are removed in the VitNet, the complex biological network will be disrupted as soon as possible with the aim of treating diseases. The GAPR method was developed to find articulation proteins in the VitNet and analyze potential therapeutic targets for vitiligo (Figure 4A). This result showed that the VitNet consisted of six layers and 160 articulation proteins (Figure 4B; Supplementary Table S6). As the network was peeled of step by step, the number of articulation proteins for each layer gradually decreased. Furthermore, we found that the articulation protein overlap rate reached 60% even if these PPIs of the VitNet were randomly removed at a rate of 50% (Figure 4C). This indicated that the GARP method had good robustness.

Subsequently, several network centrality parameters such as degree, betweenness and stress were calculated to explore the hub nodes in the VitNet (Supplementary Table S6). It was found that the distribution of the top 10 nodes (Supplementary Figure S3A) or top 100 nodes (Supplementary Figure S3B) was not commonly consistent among three network centrality parameters. These nodes that shared three types of network centrality parameters were considered as hub nodes (top 10: 5 hub nodes, top 100: 62 hub nodes). In these hub nodes, we found that articulation proteins in the VitNet had a higher coverage rate (top 10: 100%, top 100: 79%) (Supplementary Figure S3C). We also found that RGB modules (core modules) based on the GAPR method had certain consistency with MCODE method (Figure 4A; Supplementary Figure S4), but they also had certain differences. The reason is that these network centrality parameters can only identify hub nodes of initial network, and cannot further analyze the disrupted VitNet. The advantage of the GAPR method is that it can analyze all APs of the initial and disrupted networks.

**FIGURE 5:**
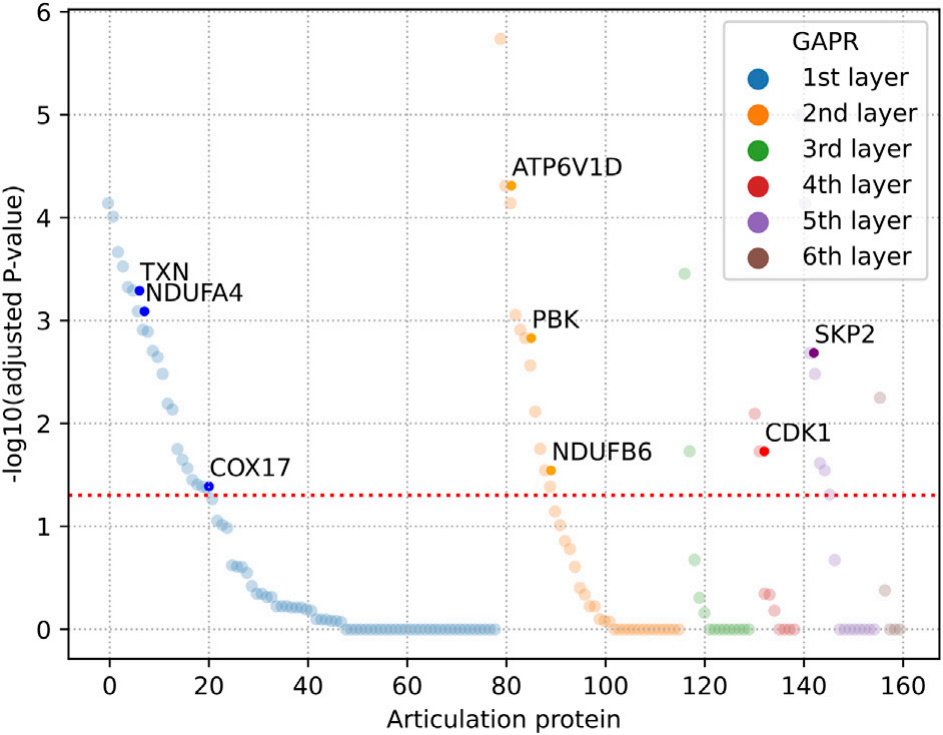
Dysregulated articulation proteins in vitiligo. The adjusted *p*-value was calculated by one-side Wilcoxon rank sum test and Benjamini-Hochberg method. GAPR: Greedy articulation points removal.

Finally, we gave priority to those articulation proteins that were highly expressed in vitiligo skin samples. The one-side Wilcoxon rank sum test was used to calculate the statistical significance of articulation proteins. The alternative hypothesis was supposed that the expression of an articulation protein in vitiligo samples could be higher than that of normal samples. There were 44 articulation proteins that were significantly high expressed in vitiligo (Figure 5; Supplementary Table S7). These articulation proteins mainly included TXN (Thioredoxin), PBK, CDK1 (Cyclin-dependent kinase 1), which may be served as potential therapeutic targets for vitiligo.

### The Multi-Target Strategy of Kaempferide for Vitiligo

In our previous works, we explored the network pharmacological mechanism of *Vernonia anthelmintica (L.)* in the treatment of vitiligo (Wang et al., 2017). The melanogenic effect of flavonoids such as kaempferide (Supplementary Figure S5A) was identified via *in vitro* and *in vivo* models (Wang et al., 2017; Wang et al., 2018; Yu et al., 2020). Some studies had also reported the similar results (Horibe et al., 2013; Xu et al., 2017; Ma et al., 2018). These studies indicated that flavonoids could play an important role in the treatment of vitiligo. However, the therapeutic mechanism of flavonoids remained unclear. In this study, we tried to apply the proteomic technology and integrate the above results to explore the underlying mechanism of kaempferide for vitiligo.

#### Proteomic Profiling of Kaempferide in B16F10 Cells

**FIGURE 6:**
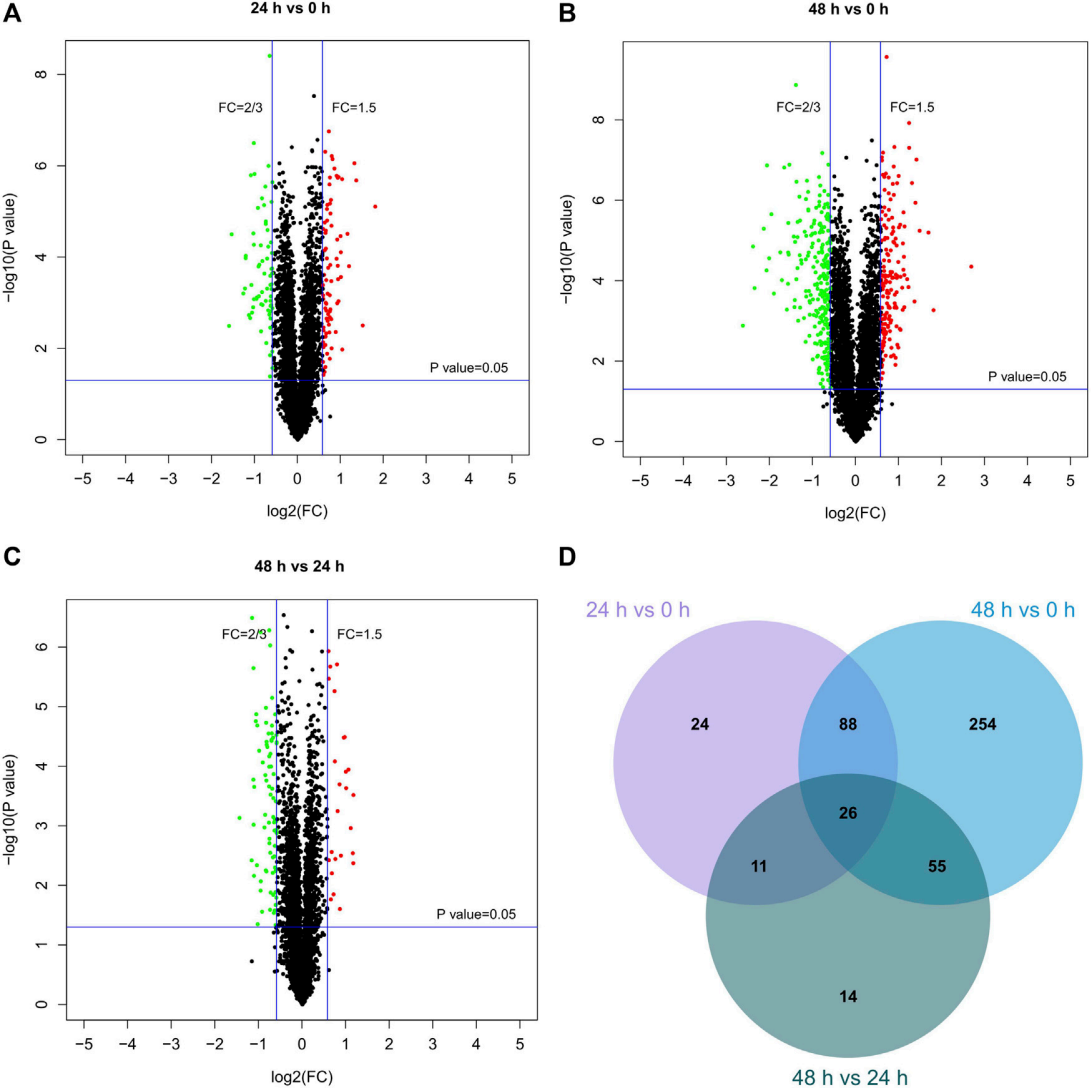
Differentially expressed proteins in different groups. **(A–C)** Volcano plots for the proteomics characterization of kaempferide in B16F10 cells. **(D)** Venn diagram of differentially expressed proteins overlapping in the comparison of different groups.

The TMT-10plex LC-MS/MS was used to analyze the proteomics characterization of kaempferide in B16F10 cells. A total of 3,707 proteins were identified and quantified (Supplementary Table S8). Principal component analysis (PCA) showed that the three groups (0, 24 and 48 h) could be clearly divided according to the first and second principal components (Supplementary Figure S5B). About 52.8 and 17.8% variation of the data could be explained by the first and second principal components, respectively. Furthermore, a total of 149 DEPs were identified in B16F10 cells in the 24 h vs. 0 h group (Figure 6A). In the 48 h *vs* 0 h (Figure 6B) and 48 h vs. 24 h (Figure 6C) groups, there were 423 and 106 DEPs, respectively. Altogether, 472 proteins were obtained by taking the union of DEPs for each group (Figure 6D; Supplementary Table S9). 26 proteins were obtained by taking the intersection of DEPs for each group (Table 1). In addition, the proteomics profiling also proved that the melanogenic pathway was activated by kaempferide (Supplementary Figure S6).

**TABLE 1:** The levels of 26 proteins at different time.

UniProt ID	Protein name	Fold change		
		24 vs. 0 h	48 vs. 0 h	48 vs. 24 h
Q60936	COQ8A	2.86	6.43	2.25
Q9D379	EPHX1	1.75	2.66	1.52
Q91VD9	NDUFS1	0.61	0.41	0.66
Q9DC70	NDUFS7	0.66	0.40	0.61
Q8VCG1	DUT	0.66	0.39	0.59
E9PVX6	MKI67	0.62	0.38	0.62
Q8BY71	HAT1	0.65	0.38	0.59
Q9CQ79	TXNDC9	0.58	0.38	0.64
Q9QWF0	CHAF1A	0.62	0.37	0.60
D3Z7B5	CIP2A	0.61	0.37	0.60
P13864	DNMT1	0.58	0.34	0.59
D3YXW1	LLPH	0.52	0.34	0.66
Q9CQ75	NDUFA2	0.60	0.34	0.56
Q80V26	IMPAD1	0.64	0.34	0.53
Q9JJ78	PBK	0.51	0.34	0.65
Q99JW7	CDK1	0.56	0.32	0.56
B1ARD6	SLFN9	0.64	0.30	0.46
Q9D1C1	UBE2C	0.47	0.27	0.56
P11157	RRM2	0.53	0.26	0.49
A0A1S6GWI4	NDUFS5	0.55	0.25	0.45
P07742	RRM1	0.50	0.24	0.48
Q3UNC9	CKS1BRT	0.64	0.24	0.37
Q3UY05	NDUFS8	0.49	0.23	0.46
Q8VDF2	UHRF1	0.43	0.20	0.46
Q3UWQ9	HMGCS1	0.34	0.19	0.55
P52431	POLD1	0.33	0.16	0.49

#### The Inhibition of CDK1 and PBK Promotes Melanogenesis

**FIGURE 7:**
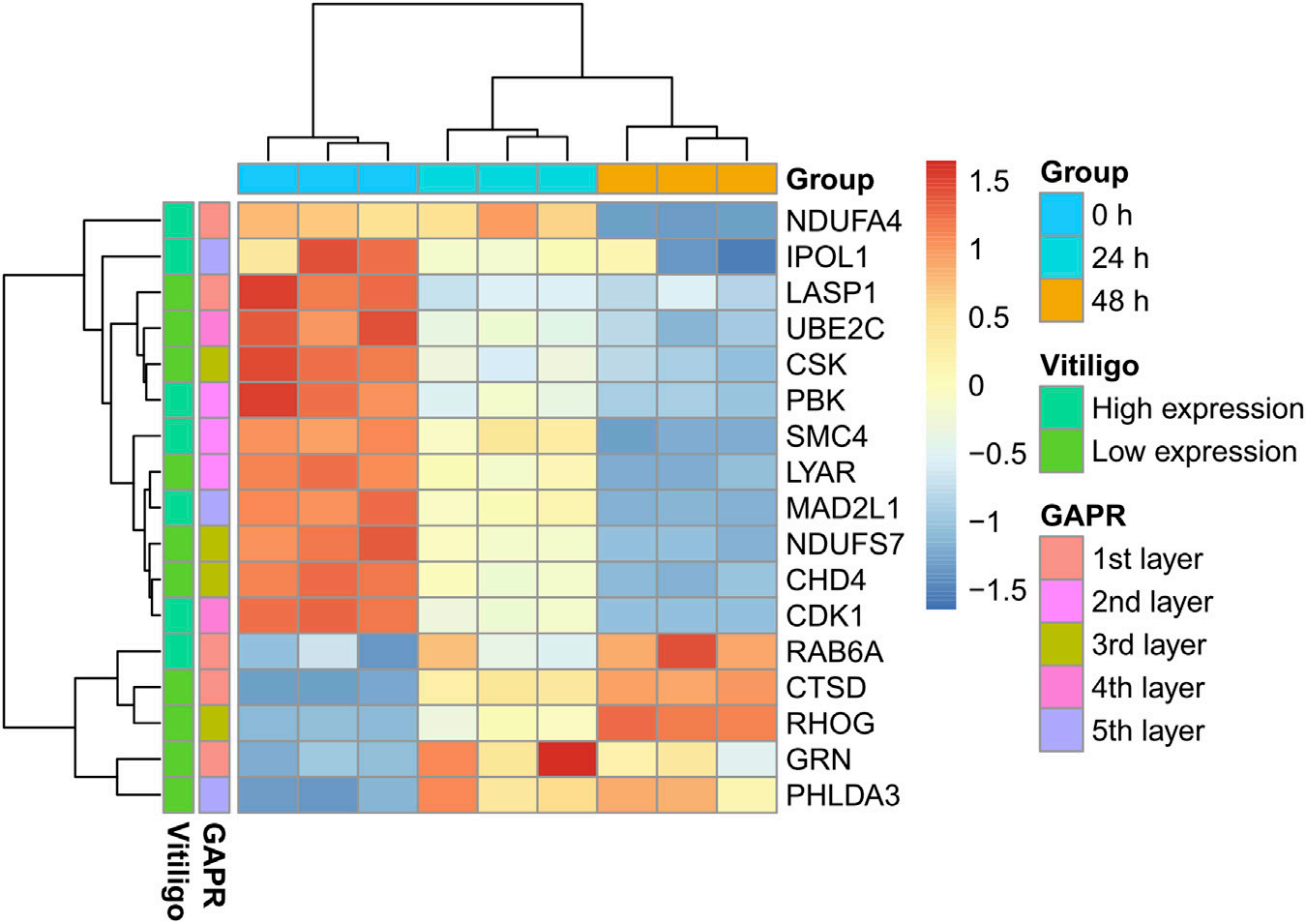
Heatmap of these 17 proteins. These proteins were the intersection of kaempferide-induced DEPs and articulation proteins based on the GAPR method. DEP: differentially expressed protein. GAPR: greedy articulation point removal.

Among these DEPs (Table 1), CDK1 and PBK were given the priority due to the intersection of kaempferide-induced DEPs and potential therapeutic targets based on the GAPR method (Figure 7). These two targets were highly expressed in vitiligo. However, in the proteomics analysis of kaempferide, the levels of these two targets significantly decreased in a time-dependent manner.

**FIGURE 8:**
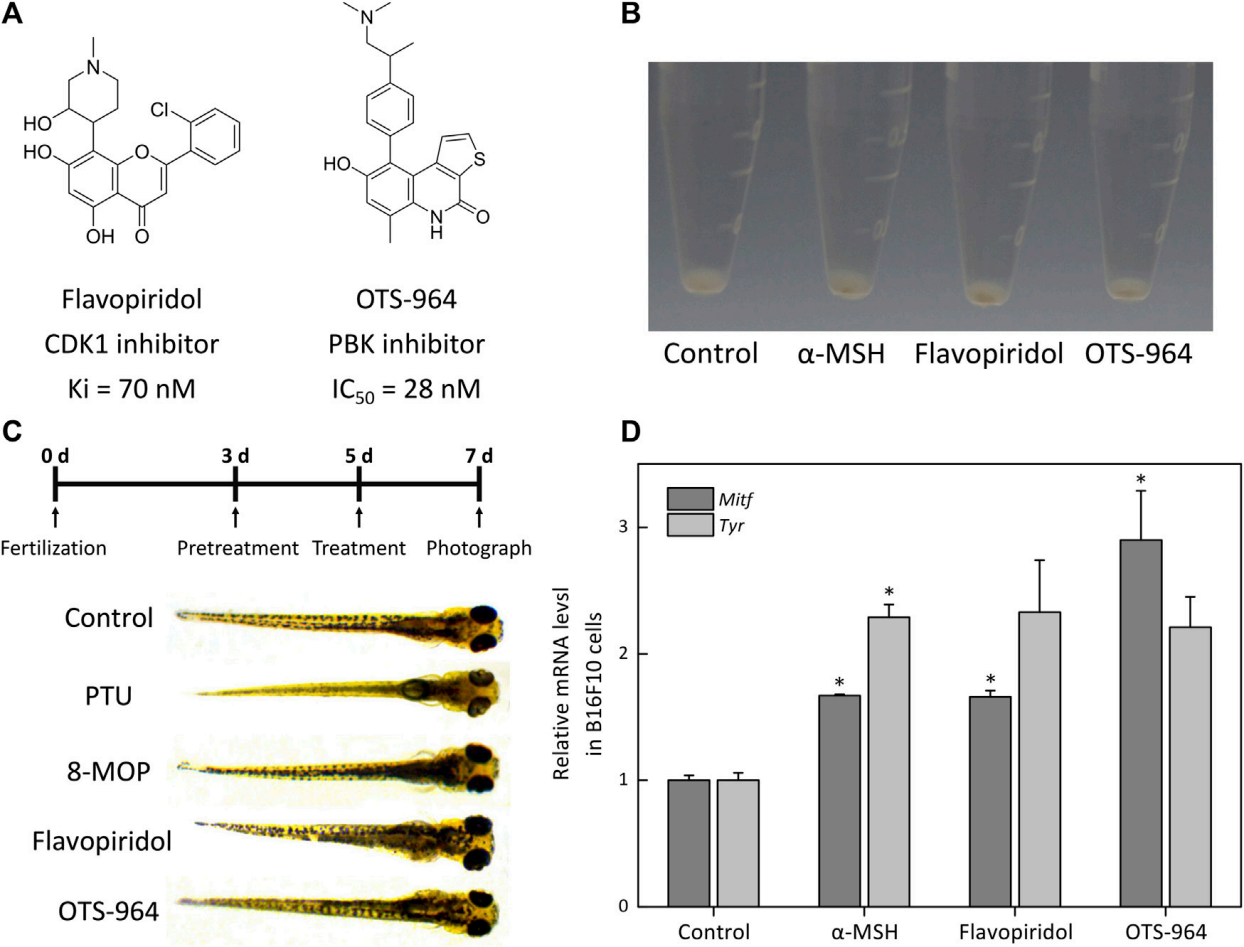
The effect of CDK1 and PBK inhibitors on melanogenesis in B16F10 cells and zebrafish. **(A)** Schematic of the chemical structure of flavopiridol (CDK1 inhibitor) and OTS-964 (PBK inhibitor). **(B)** Cells were treated with α-MSH (10 nM), flavopiridol (10 nM) and OTS-964 (10 nM) for 48 h. Cell pellets and cellular pigmentation were photographed using the PENTAX K-7. The α-MSH is a regulator of melanocyte differentiation and melanogenesis by binding to the MC1R on melanocytes. **(C)** The experimental design of the zebrafish model. Zebrafish embryos were pretreated with phenylthiourea (PTU; 200 μM) for 48 hpf. Then, these embryos were treated with methoxsalen (8-MOP; 100 μM), flavopiridol (10 nM) and OTS-964 (10 nM) for 48 hpf. PTU was used as a negative control and 8-MOP was used to a positive control. The pigmentation of the embryos was observed under a stereomicroscope. **(D)** The expressions of melanin-biosynthetic genes (*Tyr* and *Mitf*) mRNA were measured by qPCR assay. The B16F10 cells were incubated with flavopiridol (10 nM) and OTS-964 (10 nM) for 48 h.

We observed that flavopiridol (CDK1 inhibitor) and OTS-964 (PBK inhibitor) (Figure 8A) effectively promoted melanogenesis in B16F10 cells compared with α-MSH at the same concentration of 10 nM (Figure 8B). In zebrafish model, compared with control group, the melanin of zebrafish embryos pretreated with PTU was significantly reduced (Figure 8C). Then, these embryos treated with 8-MOP, flavopiridol and OTS-964 were pigmented again (Figure 8C). The expression of *Tyr* and *Mitf* mRNA also increased by the treatment of flavopiridol and OTS-964 (Figure 8D). Studies had demonstrated that PBK was phosphorylated in mitosis by CDK1 (Stauffer et al., 2017) and p38 MAPK was next phosphorylated by PBK (Abe et al., 2000). Previous studies showed that the p38 MAPK signaling pathway was associated with melanogenesis through MITF in melanocytes (Huang et al., 2013; Goding and Arnheiter, 2019). It should be noted that the p38 MAPK phosphorylation showed inconsistent expression patterns during melanogenesis (Bellei et al., 2010; Tu et al., 2012; Huang et al., 2013; Karunarathne et al., 2019; Lim et al., 2019). Our results showed that the level of MAPK14 was decreased (Supplementary Table S9). Thus, these results suggested that kaempferide induced melanogenesis through the suppression of p38 MAPK signaling pathway by inhibiting CDK1 and PBK.

#### The Regulation of Redox Proteome Promotes Melanogenesis

**FIGURE 9:**
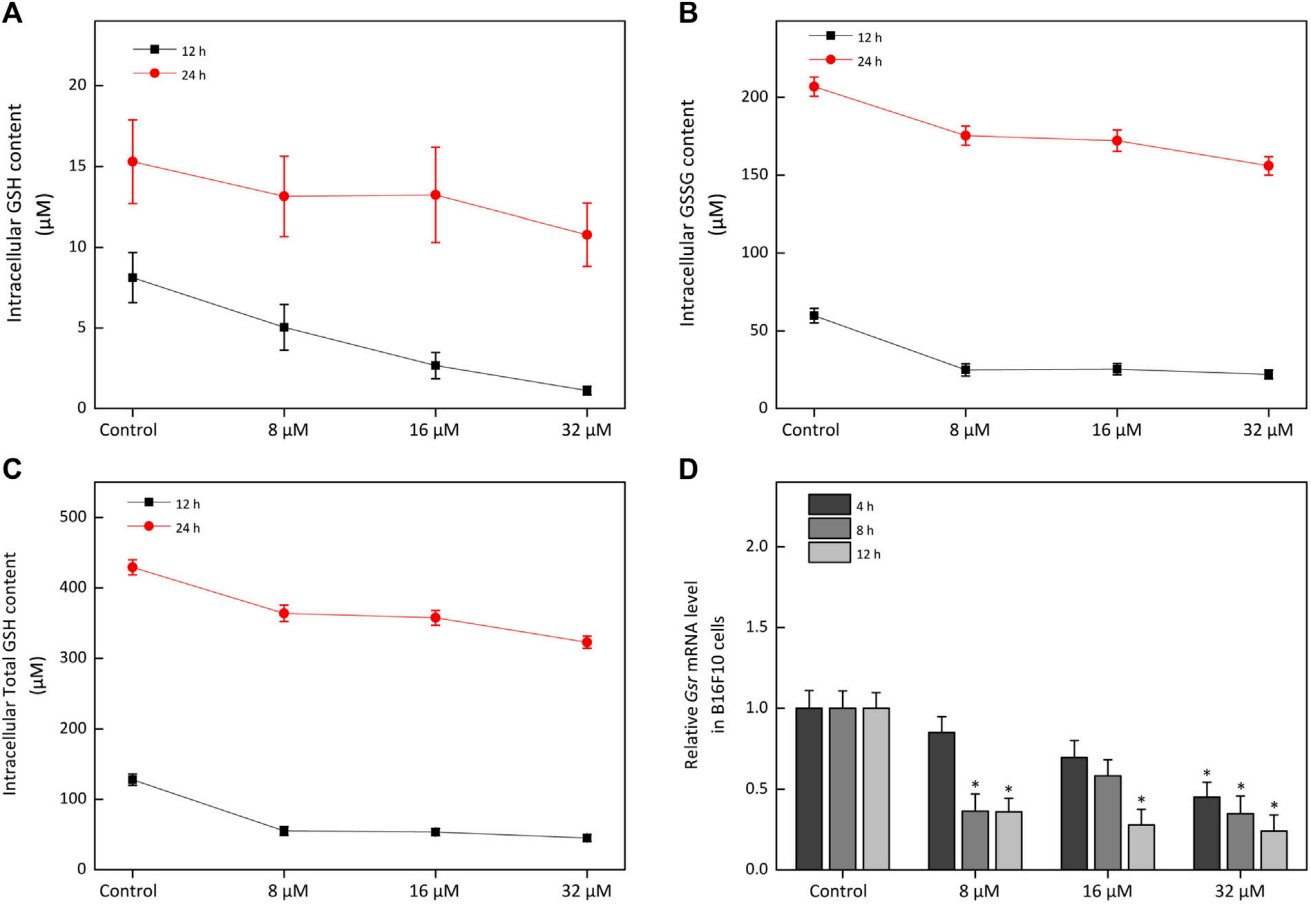
The effect of kaempferide on the glutathione metabolism in B16F10 cells. **(A–C)** The concentration of GSH, GSSG and total GSH after kaempferide treatment (8, 16 and 32 μM) in B16F10 cells for 12 and 24 h. *C*_total GSH_ = *C*_GSH_ + 2*C*_GSSG_, *C* represented concentration. **(D)** The relative expression levels to *Gsr* mRNA after kaempferide treatment (8, 16 and 32 μM) in B16F10 cells for 4, 8 and 12 h.

On the other hand, TXN was highly expressed in vitiligo skin samples (Figure 5) and controlled the cellular redox environment (Lu and Holmgren, 2014). And previous studies had demonstrated that oxidative stress was critical for the progression of vitiligo (Chang et al., 2017; Ma et al., 2018; Yi et al., 2019; Chen et al., 2021). However, there was no study to report the relationship between TXN and vitiligo, which attracted our attention. Moreover, in proteomic profiling, GO enrichment analysis showed that some DEPs were enriched in oxidation-reduction process (Supplementary Figure S7). KEGG pathway enrichment analysis also suggested that several DEPs were related to the HIF-1 and GSH metabolism pathways (Supplementary Figure S8). Previous study had reported that melanogenesis led to stimulation of HIF-1A expression and HIF-dependent attendant pathways (Slominski et al., 2014). This proteomics profiling also demonstrated this fact. Moreover, the level of RRM1 (Ribonucleoside-diphosphate reductase large subunit), RRM2 (Ribonucleoside-diphosphate reductase subunit M2) and GPX8 (Probable glutathione peroxidase 8) mainly enriched in the GSH metabolism were significantly decreased (Table 1) in a time-dependent manner. The GSH species analysis showed that kaempferide significantly decreased the intracellular content of GSH, GSSG and total GSH (Figures 9A–C). Meanwhile, the expression of *Gsr* mRNA was inhibited by kaempferide (Figure 9D). Since the GSH and TXN antioxidant systems played important roles in maintaining the cellular redox balance (Lu and Holmgren, 2014), we considered the regulation of cellular redox homeostasis as one of the multi-target strategies, which laid a foundation for further research.

**FIGURE 10:**
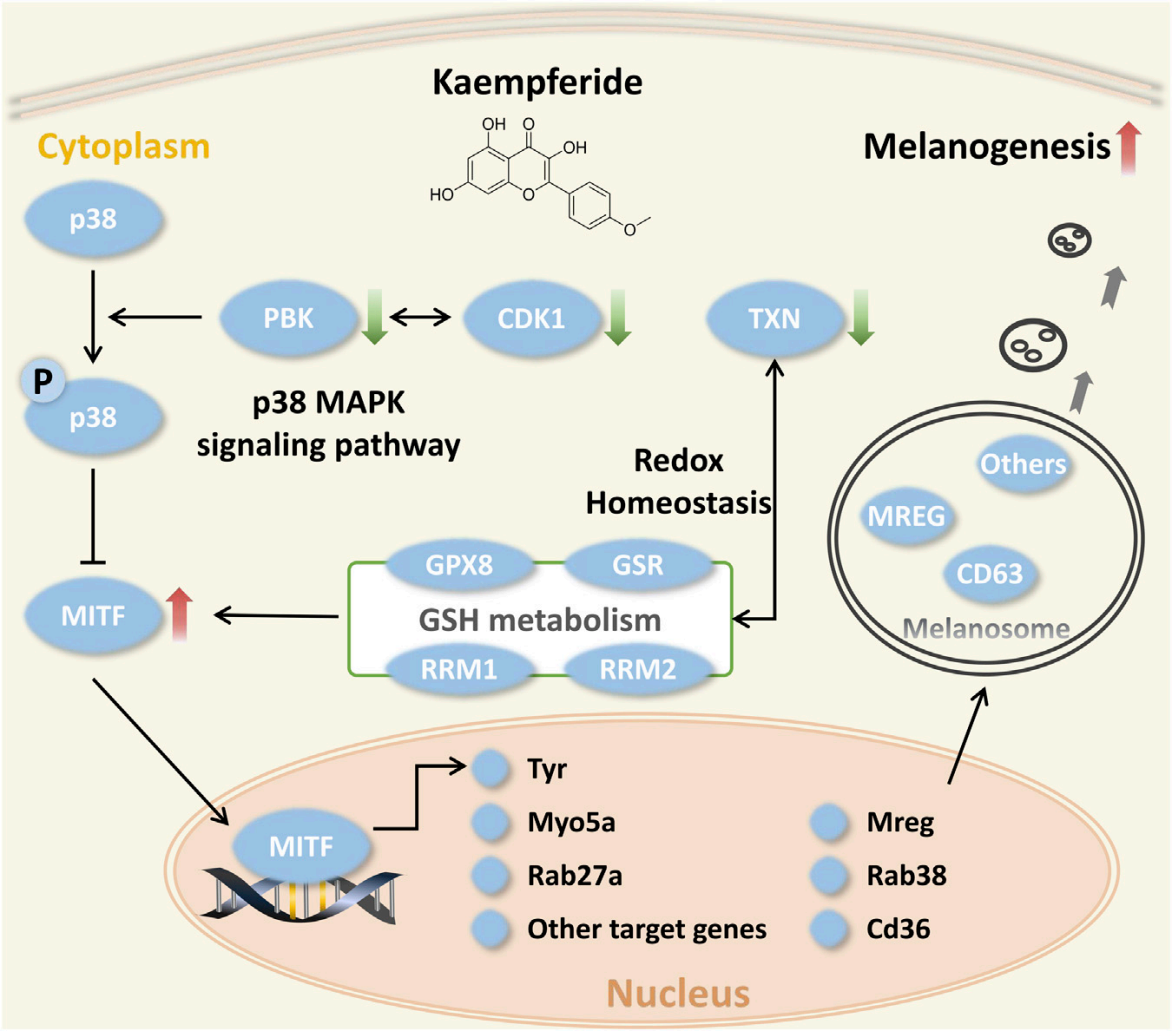
Illustration of the multi-target strategy of kaempferide for vitiligo.

By integrating the above results, we summarized that the combination of PBK, CDK1 and TXN may be the underlying mechanism of kaempferide (Figure 10) for vitiligo. It mainly included 1) the suppression of the p38 MAPK signaling pathway by inhibiting CDK1 and PBK, and 2) the modulation of cellular redox homeostasis, especially TXN and GSH antioxidant systems, for the purpose of melanogenesis. This also suggested to us that this mechanism was a novel perspective to discover novel drug candidates for vitiligo.

## Discussion

Considering the complexity of pathogenesis for vitiligo, it is difficult for single-target drugs to work. With the development of network pharmacology (Hopkins, 2008), the multi-target concept may be hopeful to explain the complex mechanism for vitiligo, especially the underlying mechanism of TCM ingredients, which is conducive to promote the development of TCMs. In previously studies, Pei et al. proposed a network pharmacology approach to uncover the multi-target mechanism of Qubaibabuqi formula for vitiligo (Pei et al., 2016). Lu et al. applied network pharmacology to analyze the multi-target mechanism of Cyclosporin A in the treatment of vitiligo (Lu et al., 2021). We had also explored the network pharmacological mechanism of *Vernonia anthelmintica (L.)* in the treatment of vitiligo (Wang et al., 2017). In addition, methoxypsoralen (8-MOP), as the first-line therapeutic drug for vitiligo, was a multi-target agent (Carbone et al., 2019). Thus, these studies indicated that the multi-target concept may offer a novel perspective for anti-vitiligo drug discovery.

In this study, through collecting transcriptome data of vitiligo and protein-protein interactome data, we designed a systematic framework to discover potential therapeutic targets for vitiligo via combining machine learning and network analysis together. Recently, Zuo et al. (2021) also proposed a similar framework to look forward with TCMs against COVID-19. With the framework, we used an interpretable machine learning method to extract important transcriptomic features for vitiligo. It was a practicality for most biological studies (Fortino et al., 2020; Shen et al., 2020; Unger et al., 2020). Among these important transcriptomic features, several top-ranked variables were closely related to the melanogenesis and immune characteristics, which was the main phenotypes of vitiligo (Picardo et al., 2015; Niu and Aisa, 2017; Pu et al., 2021; Zhang et al., 2021). Moreover, we constructed a more comprehensive VitNet and obtained 160 articulation proteins by the GAPR method. Among these articulation proteins, previous study had reported that Imatinib (ABL1 inhibitor) induced repigmentation of vitiligo lesions (Han et al., 2008). A recent study also found that PBK, CDK1 and TXN were considered as potential therapeutic targets by transcriptome and methylation analysis (Pu et al., 2021). However, whether these targets were related to melanogenesis had not been proved. In this study, we proved that by *in vitro* and *in vivo* melanogenic assays.

Furthermore, we explored the multi-target strategy for vitiligo (Figure 10) by the proteome analysis. To our limited knowledge, this is the first time that we have applied the proteome to explore the underlying mechanism of kaempferide in melanogenesis. Previous study demonstrated that alcohol extract from *Vernonia anthelmintica (L.) willd* enhanced melanin synthesis through the p38 MAPK signaling pathway (Zhou et al., 2012). However, this study did not indicate a specific therapeutic target. In this study, we predicted and experimentally validated that the multi-target composed of CDK1 and PBK played an important role in VitNet. This mechanism not only explained the melanogenesis effect of kaempferide, but also explained the molecular characteristics of complex vitiligo network. Although there was the protein-protein interaction between CDK1 and PBK, the simultaneous inhibition of multi-target in the same signaling pathway could play a synergistic role. In addition, since the GSH and TXN antioxidant systems played important roles in maintaining the cellular redox balance, the regulation of cellular redox homeostasis was considered as one of the multi-target strategies, which laid a foundation for further research. Previous study identified that microRNA-211 and its target genes (e.g. RRM2, TAOK1) regulated oxidative phosphorylation and energy metabolism and represented potential therapeutic targets for vitiligo (Sahoo et al., 2017; Goding and Arnheiter, 2019). RRM2 and TAOK1 involved in glutathione metabolism and p38 MAPK signaling pathways, respectively. This also indicated that the multi-target mechanism of kaempferide was relatively consistent with microRNA-211. Meanwhile, we noticed that the level of gene expression was not always consistent with the activity of corresponding protein. Our results could only provide the possible explanation for the multi-target mechanism of kaempferide instead of exclusive conclusion. Nevertheless, the systematic framework would be easily applied in other diseases and become a useful tool in drug discovery and development.

## Conclusion

In this study, we designed a systematic framework to discover potential therapeutic targets for vitiligo via combining machine learning and network analysis together. With the framework, we had successfully predicted and experimentally validated that some potential therapeutic targets such as CDK1 and PBK were closely related to melanogenesis, and further explored the multi-target strategy of kaempferide for vitiligo through proteomics profiling. The strategy mainly included 1) the suppression of the p38 MAPK signaling pathway by inhibiting CDK1 and PBK, and 2) the modulation of cellular redox homeostasis, especially TXN and GSH antioxidant systems, for the purpose of melanogenesis. Meanwhile, the multi-target strategy may offer a novel perspective to discover drug candidates for vitiligo. If broadly applied, the framework can become a useful tool to discover novel potential therapeutic strategies and drug candidates for other complex diseases.

## Data Availability

The datasets presented in this study can be found in online repositories. The names of the repository/repositories and accession number(s) can be found in the article/Supplementary Material
