# Supplementary material for: Development of a Multi-Target Strategy for the Treatment of Vitiligo via Machine Learning and Network Analysis Methods
Source: Front Pharmacol. 2021 Sep 15;12:754175. doi: 10.3389/fphar.2021.754175 (PMC8479195; doi:10.3389/fphar.2021.754175)
Supplement: Supplementary file 1 [file DataSheet2.DOCX]

Supporting Information

Development of a Multi-Target Strategy for the Treatment of Vitiligo via Machine Learning and Network Analysis Methods

Jiye Wang ^1,#^, Lin Luo ^2,#^, Qiong Ding ^2^, Zengrui Wu ^1^, Yayuan Peng ^1^, Jie Li ^1^, Xiaoqin Wang ^2,3^, Weihua Li ^1^, Guixia Liu ^1^, Bo Zhang ^2,3,*^, Yun Tang ^1,*^

*^1^ Shanghai Key Laboratory of New Drug Design, School of Pharmacy, East China University of Science and Technology, Shanghai 200237, China*

*^2^ Key Laboratory of Xinjiang Phytomedicine Resources of Ministry of Education, School of Pharmacy, Shihezi University, Shihezi 832002, China*

*^3^ Key Laboratory of Medicinal and Edible Plants Resources Development of Sichuan Education Department, Sichuan Industrial Institute of Antibiotics, School of Pharmacy, Chengdu University, Chengdu 610106, China*

^#^ These authors contributed equally: Jiye Wang and Lin Luo

* Correspondence: Yun Tang (E-mail address: [ytang234@ecust.edu.cn](mailto:ytang234@ecust.edu.cn)); Bo Zhang (E-mail address: [bozhang_lzu@126.com](E:/Project/Vitiligo/Result/bozhang_lzu@126.com))

# Supplementary methods

## Detail of the RPLC-MS analysis

RPLC separation was performed on an 1100 HPLC System (Agilent, USA) using an Agilent Zorbax Extend reverse-phase column (5 μm, 150 mm × 2.1 mm). Mobile phases A (2% acetonitrile in HPLC water) and B (98% acetonitrile in HPLC water) were used for reverse-phase gradient. The solvent gradient was set as follows: 0~8 min, 98% A; 8.00~8.01 min, 98%~95% A; 8.01~38 min, 95%~75% A; 38~50 min, 75~60% A; 50~50.01 min, 60~10% A; 50.01~60 min, 10% A; 60~60.01 min, 10~98% A; 60.01~65 min, 98% A. Tryptic peptides were separated at an eluent flow rate of 300 μL/min and monitored at 210 and 280 nm. These separated peptides were lyophilized for MS detection.

All analyses were performed by a Q-Exactive mass spectrometer (Thermo Fisher Scientific, USA) equipped with a Nanospray Flex source (Thermo Fisher Scientific, USA). Samples were loaded by a C18 analysis column (15 cm × 75 µm) on an EASY-nLCTM 1200 system (Thermo Fisher Scientific, USA). The flow rate was 300 nL/min and linear gradient was 90 min (from 8-100% B over 80 min; mobile phase A = 0.1% FA and B = 80% ACN/0.1% FA). Full MS scans were acquired in the mass range of 300-1600 m/z with a mass resolution of 7×10^4^ and the AGC target value was set at 1×10^6^. The ten most intense peaks in MS were fragmented with higher-energy collisional dissociation (HCD, value = 32). MS/MS spectra were obtained with a resolution of 3.5×10^4^ with an AGC target of 2×10^5^ and a max injection time of 80 ms. The Q-E dynamic exclusion was set for 15 s and run under positive mode.

# Supplementary figures

| 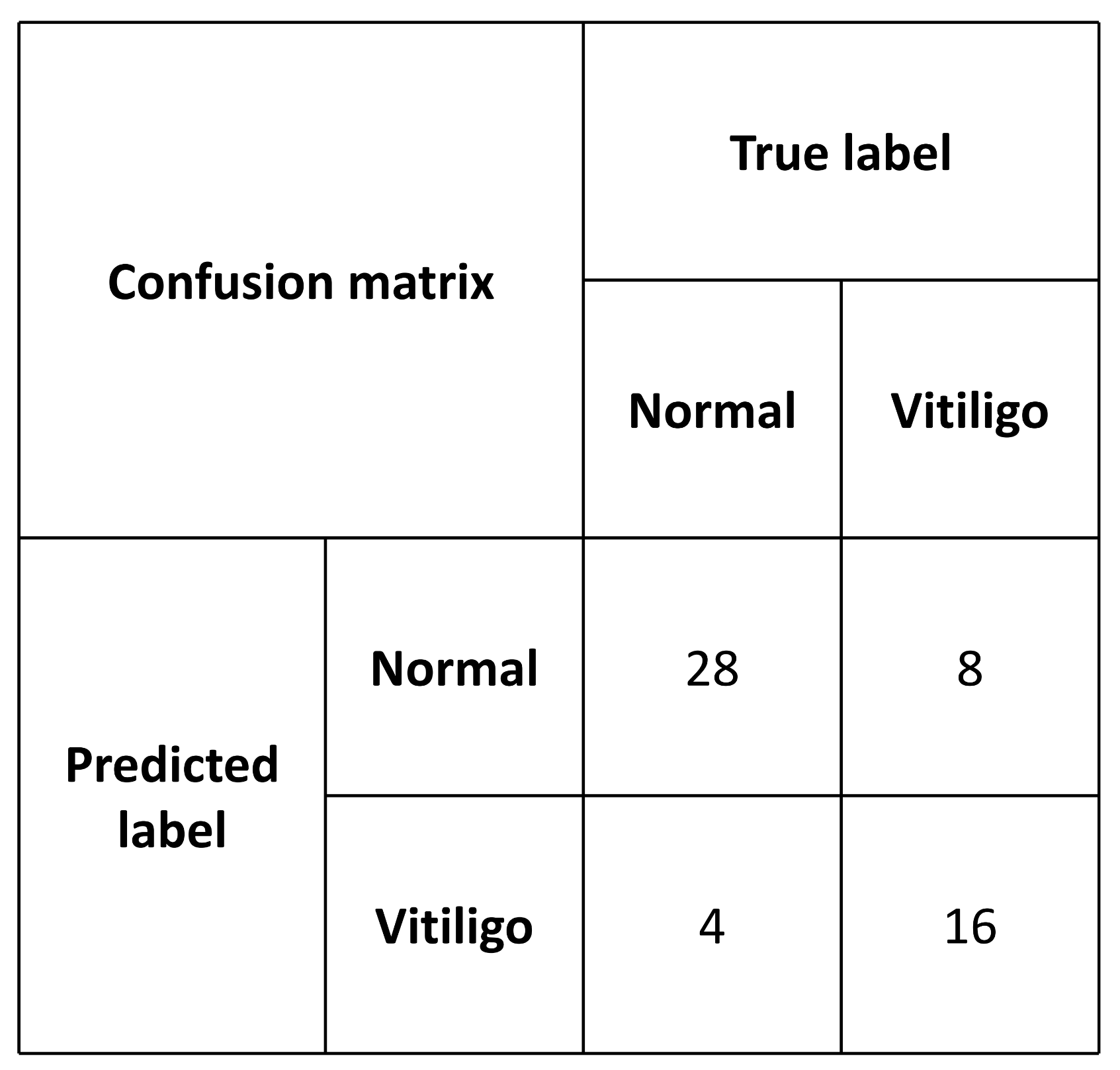 |
| --- |
| **Figure S1.** The confusion matrix of training set. The predicted label is the average results of 10-fold cross validation repeated 100 times. |

| 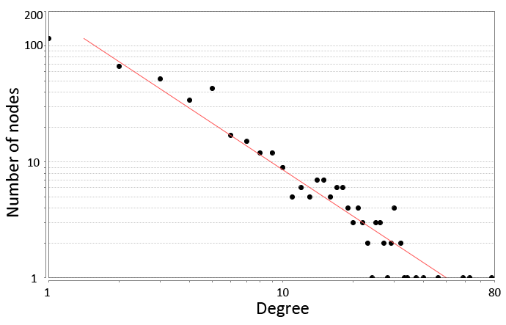 |
| --- |
| **Figure S2.** The node degree distribution of the vitiligo protein-protein interaction subnetwork (VitNet). |

| 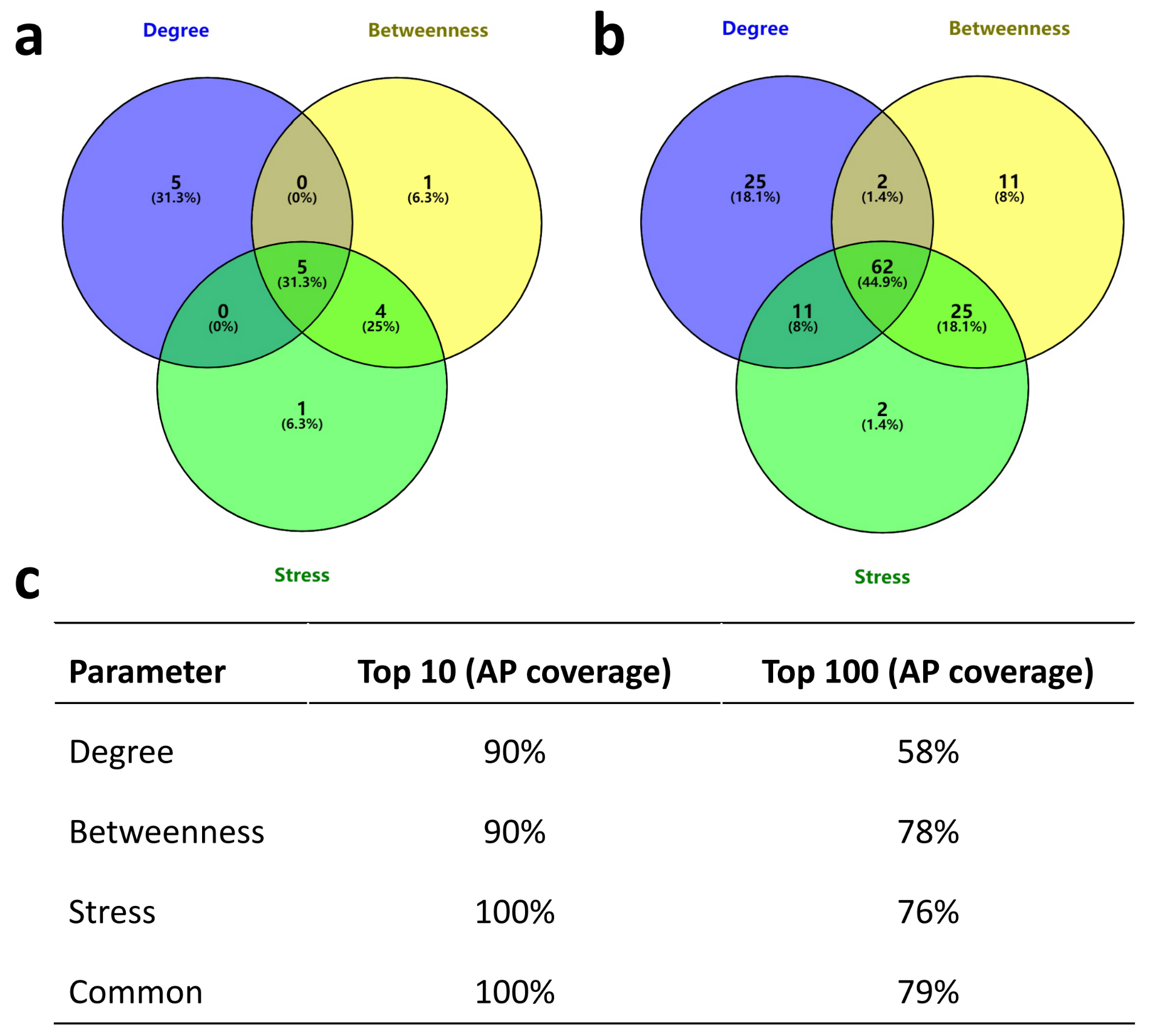 |
| --- |
| **Figure S3.** The analysis of hub nodes in VitNet by the greedy articulation points removal (GAPR) method and three network centrality parameters. |

| 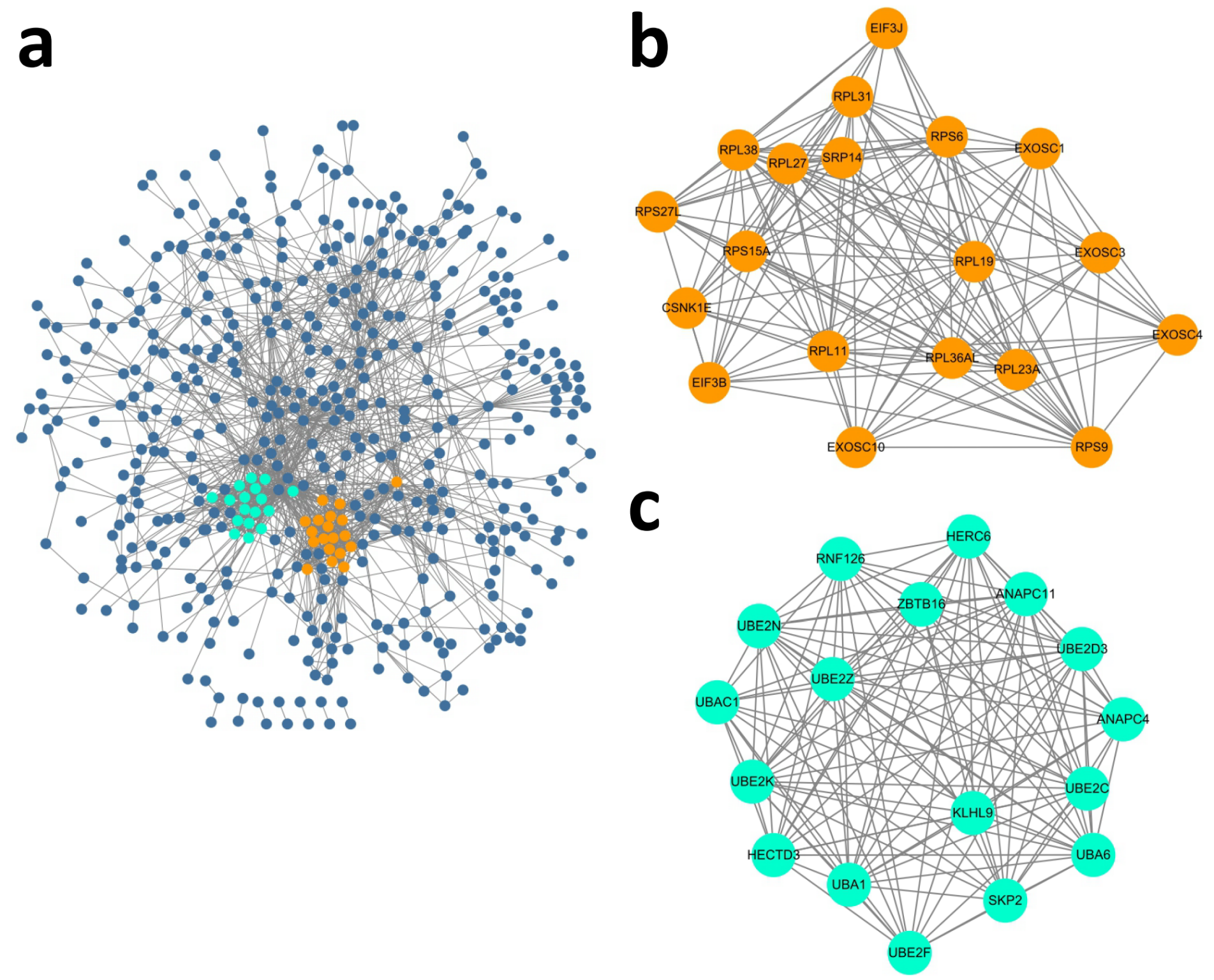 |
| --- |
| **Figure S4.** The analysis of core modules in the VitNet by the GAPR method and the Molecular Complex Detection (MCODE). |

| 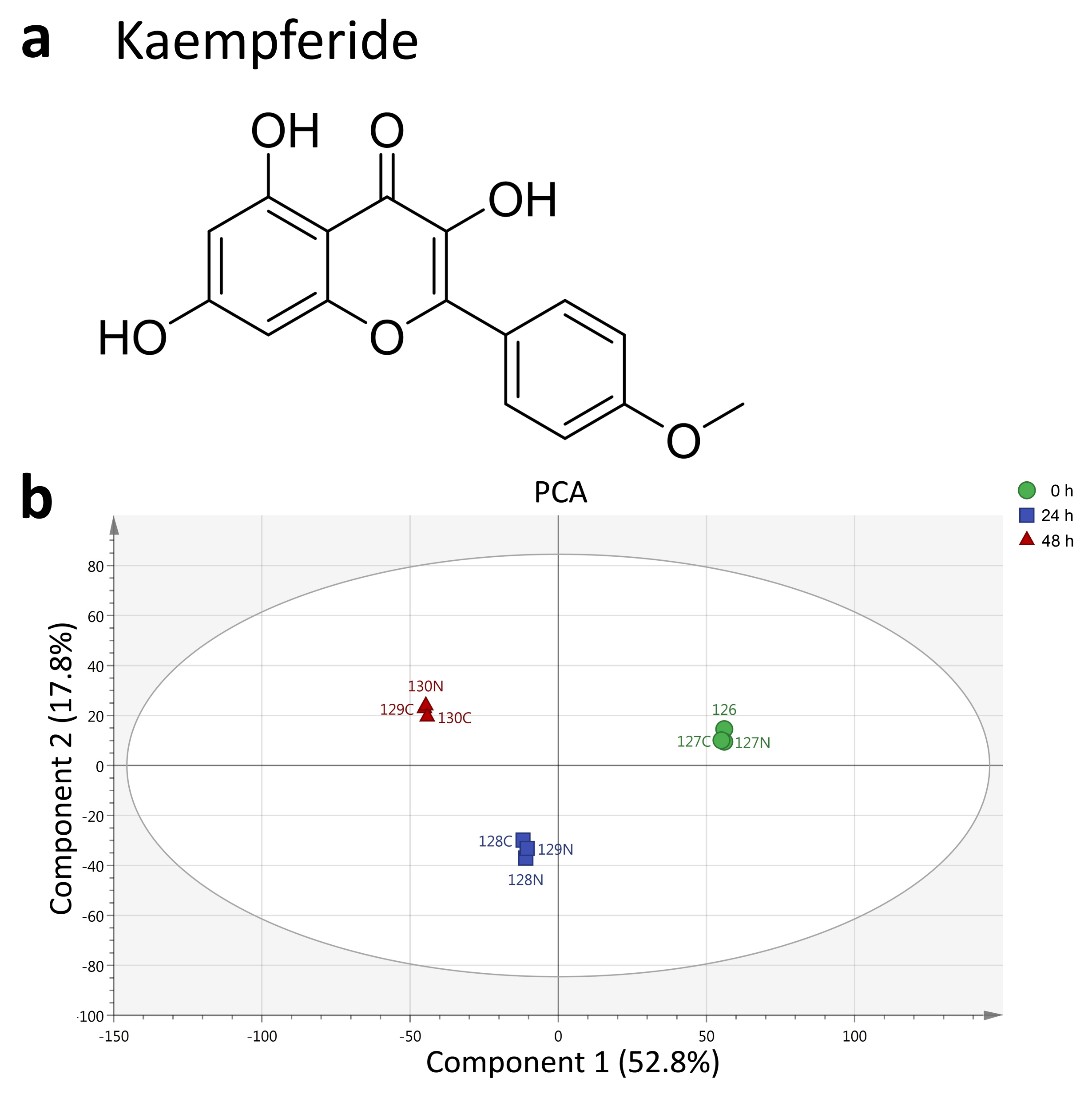 |
| --- |
| **Figure S5.** The chemical structure of kaempferide and principal component analysis (PCA) of proteomic profiling. |

| 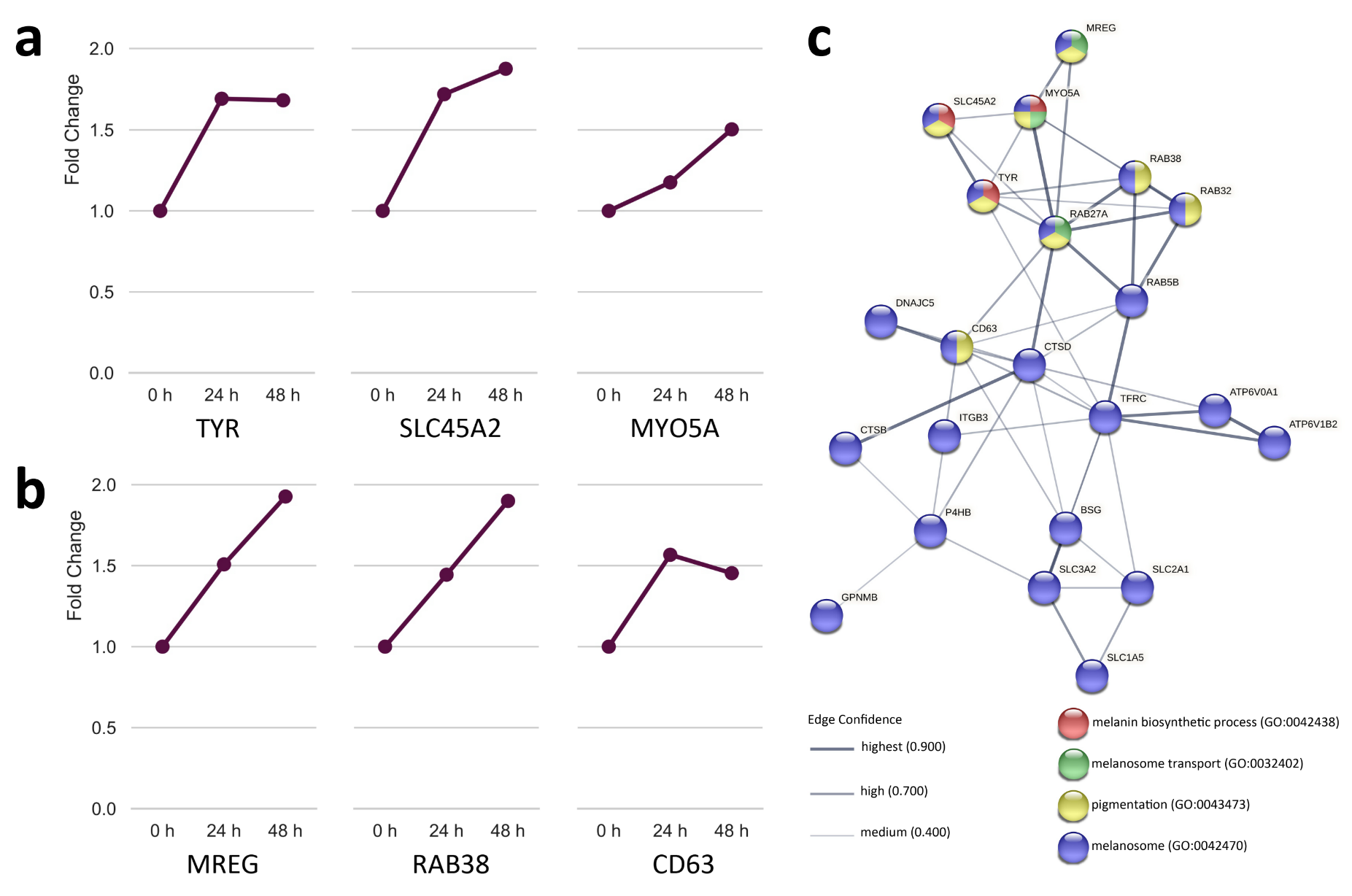 |  |  |
| --- | --- | --- |
| **Figure S6**. The levels of melanogenesis-related proteins in B16F10 cells at different time. |  |  |
| 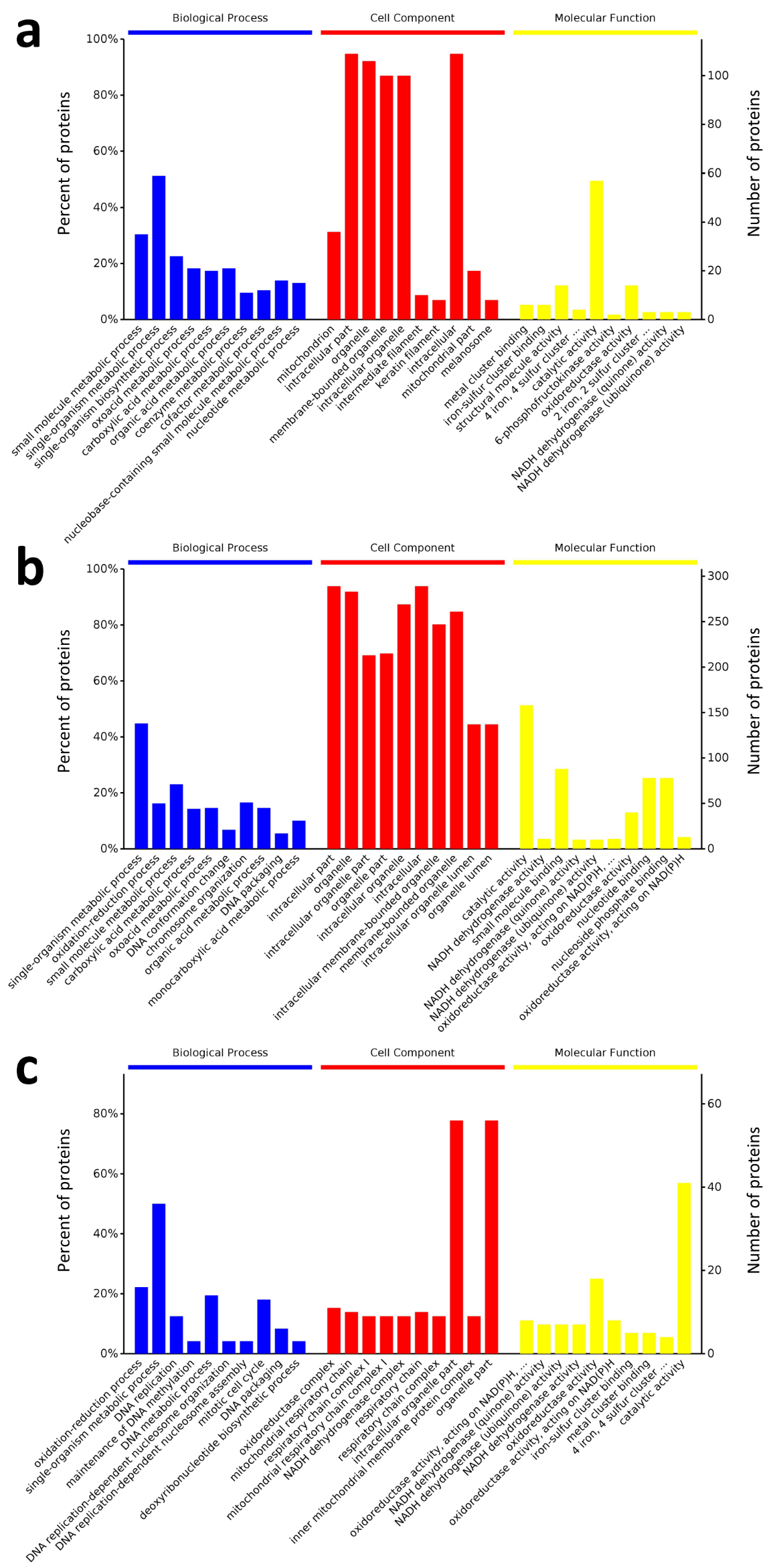 | | |
| **Figure S7.** Histogram of GO enrichment analysis with differentially expressed proteins in 24 h *vs* 0 h group (a), 48 h vs 0 h (b) and 48 h vs 24 h (c). | | |

| 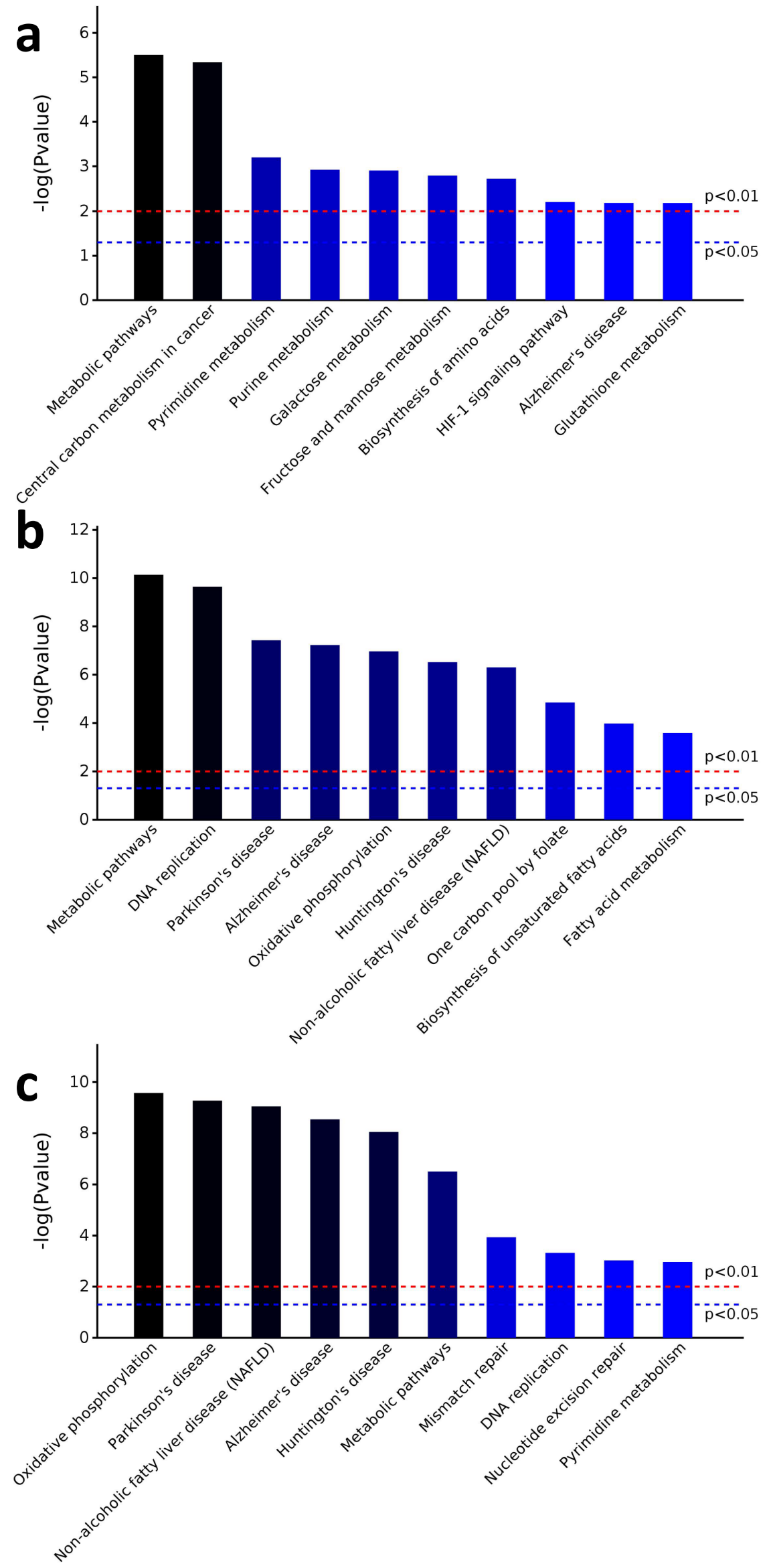 |
| --- |
| **Figure S8.** Histogram of KEGG pathway analysis with differentially expressed proteins in 24 h *vs* 0 h group (a), 48 h vs 0 h (b) and 48 h vs 24 h (c). |
